# One Year of Long-Acting Cabotegravir and Rilpivirine in People With Human Immunodeficiency Virus and Long Exposure to Antiretroviral Therapy: Data From the SCohoLART Study

**DOI:** 10.1093/ofid/ofae326

**Published:** 2024-06-20

**Authors:** Camilla Muccini, Nicola Gianotti, Sara Diotallevi, Riccardo Lolatto, Vincenzo Spagnuolo, Diana Canetti, Sabrina Bagaglio, Victoria Gordo Perez, Tommaso Clemente, Martina Bottanelli, Caterina Candela, Silvia Nozza, Antonella Castagna

**Affiliations:** Department of Infectious Diseases, IRCCS San Raffaele Scientific Institute, Milan, Italy; Department of Infectious Diseases, IRCCS San Raffaele Scientific Institute, Milan, Italy; Department of Infectious Diseases, IRCCS San Raffaele Scientific Institute, Milan, Italy; Department of Infectious Diseases, IRCCS San Raffaele Scientific Institute, Milan, Italy; Department of Infectious Diseases, IRCCS San Raffaele Scientific Institute, Milan, Italy; Department of Infectious Diseases, IRCCS San Raffaele Scientific Institute, Milan, Italy; Department of Infectious Diseases, IRCCS San Raffaele Scientific Institute, Milan, Italy; Department of Infectious Diseases, IRCCS San Raffaele Scientific Institute, Milan, Italy; Vita-Salute San Raffaele University, Milan, Italy; Vita-Salute San Raffaele University, Milan, Italy; Vita-Salute San Raffaele University, Milan, Italy; Department of Infectious Diseases, IRCCS San Raffaele Scientific Institute, Milan, Italy; Vita-Salute San Raffaele University, Milan, Italy; Department of Infectious Diseases, IRCCS San Raffaele Scientific Institute, Milan, Italy; Vita-Salute San Raffaele University, Milan, Italy

**Keywords:** HIV, antiretroviral agents, cabotegravir, rilpivirine drug combination, long-acting drugs, virological suppression

## Abstract

**Background:**

The aim of the study was to evaluate the 12-month cumulative probability of treatment discontinuation (TD) in people with human immunodeficiency virus (HIV; PWH) and a long exposure to antiretroviral therapy (ART) switching to long-acting cabotegravir and rilpivirine (CAB/RPV).

**Methods:**

SCohoLART is a single-center, prospective, cohort study designed to collect both samples and clinical data from PWH with virological suppression who switched to bimonthly long-acting CAB/RPV. TD occurred at switch to another regimen for any reason including virological failure (VF); VF was defined as HIV RNA levels ≥50 copies/mL at 2 consecutive measurements or a single HIV RNA level ≥1000 copies/mL. Results were reported as median (interquartile range [IQR]) or frequency (percentage). Cumulative probabilities of TD were estimated using Kaplan-Meier curves.

**Results:**

We evaluated 514 participants; 467 (90.9%) were male, and their median age (IQR) was 49 (40–56) years. At the time of switching, the median time from HIV diagnosis and the median duration of ART were 14.0 (IQR, 8.8–20.5) and 11.4 (7.9–17.4) years, respectively; before starting CAB/RPV, the median number of antiretroviral regimens was 3 (2–4). During a median study follow-up (IQR) of 13.1 (9.1–15.5) months, 52 PWH (10.1%) experienced TD, including 4 (0.8%) for VF. The 12-month cumulative probability of TD was 11% (95% confidence interval, 8%–14%). The main cause of TD was injection site reaction (15 participants [28.8%]).

**Conclusions:**

The 1-year cumulative probability of TD with long-acting CAB/RPV was quite low in this cohort of people with a median exposure to ART of 10 years, in whom injection site reaction was the leading cause of TD. VFs were rare during study follow-up.

## Teaser

Based on 1-year findings, the SCohoLART study showed that the strategy of switching to long-acting cabotegravir and rilpivirine is a good option for people with a prolonged history of human immunodeficiency virus infection and exposure to antiretroviral therapy.

Antiretroviral therapy (ART) has dramatically changed the course of human immunodeficiency virus (HIV) infection, reducing the incidence of AIDS-defining events and death [1, 2]. Although current oral regimens are highly effective in achieving and maintaining virological suppression, the burden of daily pill administration, treatment adherence, and infection-associated stigma remain concrete issues in the management of people with HIV (PWH) [3].

Therefore, long-acting therapy in HIV infection represents an innovative strategy to address the aforementioned unmet need, thus positively affecting the quality of life. The combination of cabotegravir (CAB), a novel integrase strand transfer inhibitor (lNSTl) and rilpivirine (RPV), a nonnucleoside reverse-transcriptase inhibitor (NNRTl), has been shown to be effective and safe in phase 3 studies when administered as a monthly or bimonthly intramuscular injection [4–7].

Considering the promising results obtained from the trials, CAB/RPV was approved for medical use in December 2020 by the European Medicines Agency and in January 2021 by the US Food and Drug Administration [8, 9]. However, results from phase 3 trials are based on populations with a short exposure to antiretroviral drugs, which may not be generalizable to PWH with a decades-long history of ART, who represent the majority of patients in clinical practice in high-income countries [4–7].

Currently, there are no data to our knowledge on PWH with >10 years of ART receiving CAB/RPV for ≥1 year; therefore, it is very important to evaluate the impact of long-acting therapy on clinical outcomes, even in those with long ART exposure. The aim of the study was to evaluate the 12-month cumulative probability of treatment discontinuation (TD) in PWH with prolonged use of ART switching to long-acting CAB/RPV therapy.

## METHODS

### Study Design and Participants

SCohoLART (cohort study of HIV-positive people treated with long-acting ART; NCT05663580) is a single-center, prospective, cohort study designed to collect both samples and clinical data from PWH on virological suppression who switched to bimonthly long-acting CAB/RPV (600/900 mg), followed up at the Department of Infectious Diseases of IRCCS San Raffaele Scientific Institute in Milan, Italy. Adult PWH with HIV RNA levels <50 copies/mL on ART were enrolled at the start of CAB/RPV (baseline); the decision to start with oral lead-in or directly with CAB/RPV injections was made after consultation with the participant's physician. People with any contraindication to the use of ≥1 long-acting drugs, according to the data sheet of the study medications (including present or past evidence of viral resistance or prior virological faiIure to NNRTls or lNSTls) [10, 11], were excluded from SCohoLART.

After initiation of CAB/RPV, HIV RNA testing were monitored at months 1, 3, and 6 and then every 6 months; the same schedule was used to monitor hepatitis B virus (HBV) DNA levels in those who are positive for antibodies to the HBV core antigen (HBcAbs). If HIV RNA was detected at >50 copies/mL, testing was repeated within the next 4 weeks. Routine blood tests were required every 6 months, including renal and metabolic assessment.

To ensure maximum adherence to scheduled injections, visits for CAB/RPV administration were managed via an app that could be freely downloaded from any mobile device, reminds patients of their appointments 48 hours in advance, and allows them to reschedule their visit for injections within the dosing period. Enrollment started on 15 July 2022, with a freeze date of 6 March 2024 for study analyses. Participants also remained in the cohort after stopping the long-acting regimen, to monitor the virological response to a rescue regimen (in case of virological failure [VF]) or toxic effects (in case of discontinuation due to adverse events).

All participants provided written informed consent before enrollment. The study protocol was approved by the Ethical Committee of IRCCS San Raffaele Scientific Institute (approved 20 June 2022; protocol 45/INT/2022). SCohoLART was conducted in accordance with the principles of Good Clinical Practice and the Declaration of Helsinki.

### Outcomes

The primary end point of the study was the cumulative proportion of people experiencing TD over 12 months. TD was considered at the occurrence of VF, defined as 2 consecutive HIV RNA levels ≥50 copies/mL or a single level ≥1000 copies/mL after initiation of long-acting CAB/RPV or at switching to another regimen for any reason. Additional secondary end points were the proportion of participants with VF and viral blips, defined as an isolated HIV RNA value ≥50 and <1000 copies/mL with adjacent values <50 copies/mL. Safety end points included renal, weight, and metabolic changes during study follow-up.

### Statistical Analysis

Participant characteristics at baseline were reported as median (interquartile range [IQR]) or frequency (percentage). Linear mixed models with random intercept and slope were calculated to estimate crude mean changes in renal and metabolic parameters; slopes (β coefficient) were reported with the corresponding 95% confidence intervals (CIs). Overall, 26 participants starting/stopping a statin during follow-up were excluded.

Cumulative probabilities (and the corresponding 95% CIs) of TD were estimated using Kaplan-Meier curves. The analyses were conducted using 2-sided *t* tests (with significance defined at α = .05) and using R Statistical Software, version 4.2.2 (R Foundation for Statistical Computing).

## RESULTS

We included 514 participants: 467 (90.9%) were male, and the median age (IQR) was 49 (40–56) years. The median time from HIV diagnosis, duration of ART, and duration of virological suppression were 14.0 (IQR, 8.8–20.5), 11.4 (7.9–17.4), and 8.6 (5.1–12.8) years, respectively. At baseline, the median CD4^+^ cell count (IQR) was 794/µL (602–994/µL), and the median CD4^+^/CD8^+^ cell ratio 0.97 (0.69–1.28), while the median nadir CD4^+^ cell count was 334/µL (214–512/µL).

The previous ART regimen was INSTI based in 313 participants (60.9%), NNRTI based in 130 (25.3%), and protease inhibitor based in 16 (3.1%); overall, 177 (34.4%) were on a 2-drug regimen, including 124 of 182 (68.1%) on dolutegravir (DTG)/lamivudine (3TC) and 45 of 182 (24.7%) on DTG/RPV. We also enrolled 122 PWH (30.2%) who were positive for HBcAb and negative for HBV surface antigen (HBsAg). At baseline, 55 participants (10.7%) received oral lead-in with CAB/RPV before starting injections. The main participant characteristics are reported in Table 1.

**Table 1.** Participant Characteristics at Time of Switch to Cabotegravir and Rilpivirine

Characteristic	Participants, No. (%)^a^ (N = 514)
Age, median (IQR), y	49 (40–56)
Male sex	467 (90.9)
HIV risk group	
Men who have sex with men	364 (70.8)
Heterosexuals	58 (11.3)
People who inject drugs	11 (2.1)
Other	81 (15.8)
Time from HIV diagnosis, median (IQR), y	14.0 (8.8–20.5)
Duration of ART, median (IQR), y	11.4 (7.9–17.4)
Previous AIDS diagnosis	52 (10.3)
HIV RNA	
Target not detected	324 (63.0)
<50 copies/mL	190 (37.0)
CD4^+^ cell count, median (IQR), cells/µL	794 (602–994)
Nadir CD4^+^ cell count	
>200/µL	389 (76.4)
≤200/µL	120 (23.6)
CD8^+^ cell count, median (IQR), cells/µL	850 (643–1114)
HBcAb positive	122 (30.2)
HCV Ab positive	56 (11.2)
Duration of ART regimen at CAB/RPV switch, median (IQR), y	3.4 (2.2–5.4)
No. of drugs in ART regimen at CAB/RPV switch, median (IQR)	3.00 (2.00–3.00)
Type of ART regimen at CAB/RPV switch	
2 NRTIs + 1 PI	14 (2.8)
2 NRTIs + 1 NNRTI	124 (24.6)
2 NRTIs + 1 INSTI	179 (35.5)
1 NRTI + 1 INSTI	124 (24.6)
1 INSTI + 1 NNRTI	45 (8.8)
PI monotherapy	5 (1.0)
Other	13 (2.5)

Abbreviations: ART, antiretroviral therapy; CAB/RPV, cabotegravir and rilpivirine; HBcAb, antibodies to hepatitis B virus core antigen; HCV Ab, hepatitis C virus antibody; HIV, immunodeficiency virus; INSTI, integrase strand transfer inhibitor; IQR, interquartile range; NNRTI, nonnucleoside reverse-transcriptase inhibitor; NRTI, nucleoside reverse-transcriptase inhibitor; PI, protease inhibitor.

^a^Data represent no. (%) of participants unless otherwise specified.

**Figure 1.**
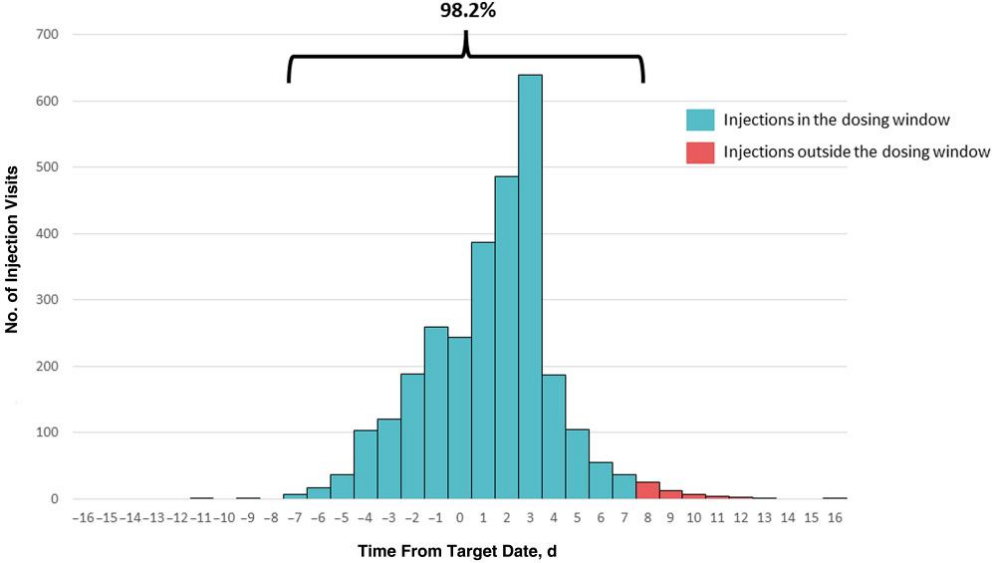
Proportion of injection visits occurring within the dosing window.

During the SCohoLART study, 3438 ventrogluteal injections were administered. Excluding 514 first injections, 2924 injections were performed through the follow-up, and 2870 (98.2%) of the scheduled injections were administered within the dosing window of ±7 days; in addition, 2 (0.01%) were received >7 days before the scheduled injection date and 52 (1.8%) >7 days after the scheduled injection date, as shown in Figure 1.

During a median time study follow-up time (IQR) of 13.1 (9.1–15.5) months, 52 PWH (10.1%) experienced TD; the median time to TD was 5.1 (2.7–8.9) months. The main cause of TD was injection site reaction (ISR) in 15 participants (28.8%), followed by toxic effects (excluding gastrointestinal and central nervous system effects) in 10 (19.2%). All the reasons for TD are listed in Table 2.

**Table 2.** Reasons for Discontinuation of Long-Acting Treatment

Reason for Discontinuation	Participants, No. (%) (n = 52)
Injection site reaction	15 (28.8)
Other toxic effects^a^	10 (19.2)
Other causes^b^	8 (15.4)
Participant's wish/decision	5 (9.6)
Virological failure	4 (7.7)
Central nervous system toxic effects	4 (7.7)
Gastrointestinal tract toxic effects	3 (5.8)
Hypersensitivity reaction/allergy	2 (3.8)
Death^c^	1 (1.9)

^a^Other toxic effects included pyrexia in 3 participants; fatigue, joint stiffness, and weight gain in 2 each; and polyarthralgia in 1.

^b^Other causes included personal commitments incompatible with scheduled injection visits in 2 participants and transfer to another country, hepatitis B virus reactivation, pregnancy, viral blip, enrollment in another study protocol, and chemotherapy-induced thrombocytopenia in 1 each.

^c^One participant died of complications secondary to a metastatic biliopancreatic adenocarcinoma.

Overall, 1 of 122 participants positive for HBcAb discontinued treatment due to the acute exacerbation of chronic hepatitis B: at baseline, HBsAg and HBV DNA were negative for 6 years; the HBV genotype was A both before and after the start of the CAB/RPV regimen. After CAB/RPV interruption, the participant restarted the previous ART regimen with bictegravir (BIC)/emtricitabine (F)/tenofovir alafenamide (TAF), which had been taken for almost 3 years before the injections; transaminase levels normalized after 4 weeks, and HBV DNA returned to negative at 24 weeks.

One participant died of complications secondary to a metastatic biliopancreatic adenocarcinoma, diagnosed 2 months after the start of long-acting therapy and considered unrelated to current antiretroviral drugs.

Among participants who experienced TD, 4 (0.8%) had VF and switched to CAB/RPV from an INSTI-based regimen (2 from BIC/F/TAF, 1 from DTG/3TC/abacavir, and 1 from DTG/RPV). The first participant met VF criteria at month 5 with HIV RNA levels of 371 copies/mL at the first and 436 copies/mL at the second measurement; virological suppression was achieved 4 weeks after initiation of darunavir/cobicistat (DRV/c)/F/TAF.

At month 5, the second participant's viral load reached 34 300 copies/mL, leading to discontinuation of the long-acting regimen in favor of DRV/c/F/TAF; an HIV RNA level <50 copies/mL was obtained again 32 weeks after the therapeutic switch. The third participant had an initial HIV RNA of 636 copies/mL at month 6, then 66 500 copies/mL 2 weeks later; after the stopping of CAB/RPV, DRV/c/F/TAF was started, and the viral load after 20 weeks was undetectable. Finally, at month 3, the fourth participant had 2 consecutive HIV RNA values >50 copies/mL, specifically 276 and 55 copies/mL; virological suppression was achieved 1 week after the switch to BIC/F/TAF.

The first 3 participants had a sexually transmitted infection concomitant with VF, 2 with gonococcal urethritis and 1 with primary syphilis, which were adequately treated at the time of symptom onset. Moreover, these 3 participants had received an NNRTI-based regimen in the past (RPV-containing ART in 2 people) for ≥2 years and maintained virological suppression over the course of oral therapy; they had resistance-associated mutations to RPV and INSTIs on genotypic resistance testing (GRT) performed on both RNA and DNA at failure. For these participants, pretreatment genotypes were not available.

**Figure 2.**
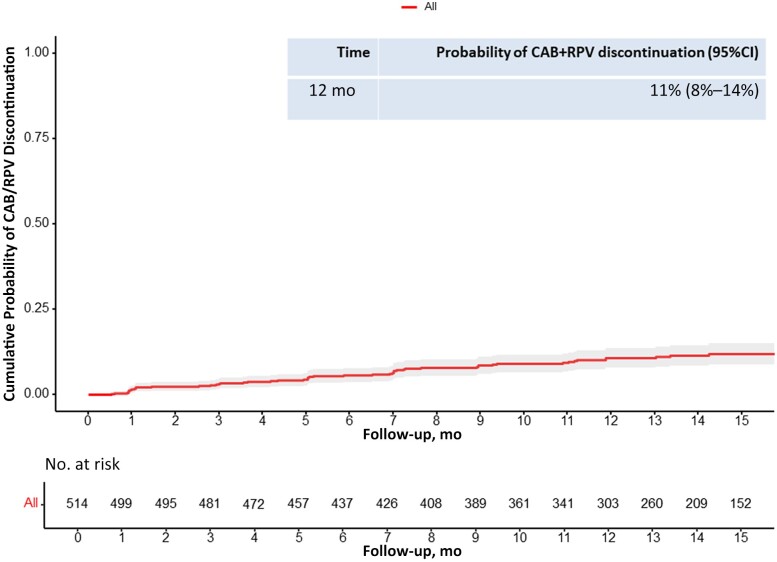
Cumulative probability of treatment failure. Abbreviations: CAB/RPV, cabotegravir and rilpivirine; CI, confidence interval.

All the PWH experiencing VF received injections within the dosing window; detailed characteristics of participants with VFs are summarized in Table 3. The cumulative probabilities of TD were 3% (95% CI, 2%–5%) at month 3, 6% (4%–8%) at month 6, and 11% (8%–14%) at month 12, as illustrated in Figure 2.

**Table 3.** Characteristics of Participants Experiencing Virological Failure

Participant/Sex/Age, y	Year of HIV Diagnosis	Subtype	BMI^a^	Pre–CAB/RPV Regimen	HIV RNA at VF, Copies/mL	RPV at VF, ng/mL	STI at VF	GRT at VF^b^	Post–CAB/RPV Regimen	HIV RNA After CAB/RPV Switch, Copies/mL
1/Male/50	2005	B	22.2	DTG/ABC/3TC	371; 436	121.8	Gonococcal urethritis	NNRTI (RNA): E138A NNRTI (DNA) E138EA INSTI (RNA): E138EK, Q148R; INSTI (DNA): Q148QR	DRV/c/F/TAF	<50 (after 4 wk)
2/Male/41	2007	B	25.7	DTG/RPV	34 300	170.5	Gonococcal urethritis	NNRTI (RNA) K101PQ, E138A INSTI (RNA) E138K, Q148R	DRV/c/F/TAF	<50 (after 32 wk)
3/Male/56	2012	B	24.6	BIC/F/TAF	636; 66 500	50.5	Primary syphilis	NNRTI (RNA) K101E, E138A NNRTI (DNA) K101KE, E138EA; INSTI (RNA/DNA): E157Q	DRV/c/F/TAF	<50 (after 20 wk)
4/Male/57	2018	B	25.3	BIC/F/TAF	276; 55	70.8	/	WT for NNRTIs and INSTIs	BIC/F/TAF	<50 (after 1 wk)

Abbreviations: 3TC, lamivudine; ABC, abacavir; BIC, bictegravir; BMI, body mass index; CAB/RPV, cabotegravir and rilpivirine; DRV/c, darunavir/cobicistat; DTG, dolutegravir; F, emtricitabine; GRT, genotypic resistance testing; HIV, human immunodeficiency virus; INSTI, integrase strand transfer inhibitor; NNRTI, nonnucleoside reverse-transcriptase inhibitor; STI, sexually transmitted infection; TAF, tenofovir alafenamide; VF, virological failure; WT, wild type.

^a^BMI calculated as weight in kilograms divided by height in meters squared.

^b^Pretreatment GRT was available only for participant 4: HIV was WT for both NNRTIs and INSTIs. All participants received injections within the dosing window.

At discontinuation of long-acting therapy, 16 participants (30.8%) started DTG/3TC, 15 (28.8%) BIC/F/TAF, 7 (13.5%) RPV/F/TAF, 4 (7.7%) DTG/RPV, 4 (7.7%) DRV/c/F/TAF, and 5 (9.6%) other drug regimens. After switching from long-acting therapy with CAB/RPV, all participants maintained or achieved HIV RNA levels <50 copies/mL. The proportion of PWH with viral blips throughout the study was 4.5% (23 of 514); before starting injections, 11 (47.8%) were on an INSTI plus 2 nucleoside reverse-transcriptase inhibitors, 7 (30.4%) on an NNRTI plus 2 nucleoside reverse-transcriptase inhibitors, and 5 on dual therapy (21.7%). VF did not develop in any participants with viral blips.

At CAB/RPV initiation, the median creatinine level (IQR) was 1.1 (1.0–1.2) mg/dL, and median estimated glomerular filtrate rate (eGFR; obtained using the Chronic Kidney Disease Epidemiology Collaboration [CKD-EPI] equation) was 80 (70–89) mL/min/1.73 m^2^. Overall, the mean changes (95% CIs) in creatinine level and eGFR were −0.1 mg/dL/y (−.1 to −0.1) and +2.8 (1.9–3.8) mL/min/1.73 m^2^ (both *P* < .001).

Regarding the metabolic profile, at baseline the median total cholesterol (TC) level (IQR) was 180 (159–202) mg/dL; the median high-density lipoprotein (HDL) cholesterol (HDL-C) level, 48 (41–57) mg/dL, and the median low-density lipoprotein cholesterol level, 113 (96–136) mg/dL; moreover, the median TC/HDL-C ratio was 3.7 (3.1–4.4). The median triglyceride level (IQR) was 99 (76–136) mg/dL, while median glucose level and homeostatic model assessment for insulin resistance were 89 (82–96) mg/dL and 1.7 (1.2–2.7), respectively; furthermore, the median weight was 76 (69–85) kg, and the median body mass index (BMI) 25 (23–27) (calculated as weight in kilograms divided by height in meters squared). In participants switching to long-acting therapy, the mean changes in HDL-C and TC/HDL-C ratio were +3.0 mg/dL/y (95% CI, 1.9–4.1) and −0.2/y (−.3 to −0.1), respectively (both *P* < .001). Other changes are described in Table 4.

**Table 4.** Crude Mean Changes in Renal, Weight, and Metabolic Parameters After Switch to Cabotegravir and Rilpivirine

Variable	Overall Crude Mean Change (Slope) per Year (95% CI)	*P* Value
Creatinine, mg/dL	−0.1 (−.1 to −.1)	<.001
eGFR, mL/min/1.73 m^2^	2.8 (1.9–3.8)	<.001
Weight, kg	0.4 (−.2 to 1.0)	.15
BMI^a^	0.1 (0–.3)	.12
Glucose, mg/dL	0.2 (−2.1 to 2.4)	.88
HOMA index	0.1 (−.2 to .5)	.45
Triglycerides, mg/dL	−3.3 (−8.1 to 1.5)	.18
TC, mg/dL	0.5 (−2.4 to 3.3)	.75
HDL-C, mg/dL	3.0 (1.9–4.1)	<.001
LDL-C, mg/dL	0.5 (−1.9 to 2.9)	.70
TC/HDL-C ratio	−0.2 (−.3 to −.11)	.001

Abbreviations: BMI, body mass index; CI, confidence interval; eGFR, estimated glomerular filtrate rate; HDL, high-density lipoprotein cholesterol; HOMA, homeostasis model assessment insulin resistance; LDL-C, low-density lipoprotein cholesterol; TC, total cholesterol.

^a^BMI calculated as weight in kilograms divided by height in meters squared.

## DISCUSSION

In this cohort of PWH with a median exposure to ART of 10 years, the cumulative probability of TD during the first year of CAB/RPV was quite low, with few cases of VF, consistent with findings from previous trials. In the phase 3 SOLAR and FLAIR trials, rates of discontinuation for long-acting therapy at 48 weeks were slightly less than 10% (43 of 447 [9.6%] and 25 of 283 [8.8%], respectively), while in real-life studies TD rates are between 12% and 13% [12, 13]. The reasons for stopping regimen were heterogeneous; moreover, grade 4 drug-related adverse events were anecdotal [4–7]. ISRs were the most common clinical event reported in trials, mostly mild or moderate, but only a small number of patients discontinued long-acting therapy due to the appearance of pain, nodules, or edema. Indeed, the proportion of participants interrupting CAB/RPV for ISRs in most phase 3 trials (ATLAS, ATLAS-2 M, and FLAIR) was about 1% at 48 weeks [5–7, 12–16]; our results are comparable to those of the SOLAR study, which reported 10 of 447 discontinuations (2.2%) for ISRs in the first year of long-acting treatment [4].

Although our findings are in line with those of clinical trials, PWH enrolled in the SCohoLART study have different characteristics; in fact, our participants had a longer duration of ART exposure and a higher median age, with about 15% of PWH >60 years old included in the analysis. Phase 3 clinical trials have included PWH with a short history of ART; the median duration of antiretroviral drug use did not exceed 2.5 years or participants could not have received >2 antiretroviral regimens since HIV diagnosis to be included in the study [4–7]. In addition, the median age of participants receiving long-acting therapy in the clinical trials was approximately 7–15 years younger than in our study, with a maximum of 42 years [4–7].

Older people are typically underrepresented in phase 3 studies. They are a category to be considered because they have an increased incidence of comorbid conditions, and the impact of new antiretroviral drugs should be carefully evaluated in this population. Although real-world data have described PWH with characteristics more similar to those in the SCohoLART study than in the trials, there are still no published data on participants >50 years old with at least a decade of ART history receiving CAB/RPV for >1 year. Therefore, it was crucial to observe a VF rate <1% even in a population with long-term antiretroviral exposure, with previous therapeutic switching studies reporting VFs ranging between 1% and 2% [4–7, 12–18]; this consistency was also found in the percentage of viral blips recorded [19].

Thus, it is important to identify PWH at highest risk for VF. According to a pooled analysis of 1651 participants, having ≥2 of the following significantly increased the risk of experiencing treatment failure: the presence of RPV resistance-associated mutations, HIV subtype A6/A1, and BMI ≥30 at the time of switching to CAB/RPV [20]. Indeed, higher BMI has been proved insufficient to decrease the efficacy of CAB/RPV in virologically suppressed individuals at the start of long-acting therapy, as shown in the OPERA cohort, where no differences in VF were found between 1022 PWH with a BMI <30 and 450 with a BMI ≥30 [21]. In our study, all 4 participants who experienced VF had a BMI <30 and were subtype B, and only 1 had an RPV concentration close to the threshold target of 50 ng/mL at treatment failure.

We also observed the concomitance of a sexually transmitted infection in 3 of the 4 participants in whom VF developed; the numbers are clearly too small to investigate a correlation, but we know that the onset of syphilis and gonorrhea can lead to viral blips in people who are virologically suppressed [22, 23]. Furthermore, 4 of 4 switched from an INSTI-based regimen and 3 of 4 previously received an NNRTI-based regimen, without ever recording a treatment failure and thus always maintaining undetectable viremia. Thus, VF with injectable CAB/RPV can also occur in the absence of known risk factors at baseline, similarly to what has been described in 3 of 5 PWH with treatment failure reported by van Welzen et al [24].

In our study, of the 3 participants who developed resistance-associated mutations to INSTIs and NNRTIs at VF, none had available pretreatment GRT. In fact, in people without failure of previous therapy containing INSTIs and/or NNRTIs, it is not currently indicated to require GRT on peripheral DNA before starting a long-acting CAB/RPV regimen. However, there are mutations in the reverse transcriptase gene, such as those at position 138 (particularly E138A), that can occur as natural polymorphisms in about 3%–4% of PWH, especially if harboring non–B subtype viruses [25]. Because mutations at codon 138 (K, Q/G, R) are associated with reduced susceptibility to RPV [26], GRT on peripheral DNA may be considered in patients without historical GRT to better identify candidates for long-acting therapy and to prevent VFs in those harboring drug-resistant variants.

In the SCohoLART study, 1 participant discontinued treatment due to the acute exacerbation of chronic hepatitis B, which resolved after starting BIC/F/TAF. Although cases of HBV reactivation during dual therapy, including CAB/RPV, are very rare in the literature [26, 27], it is important to check HBV serology before initiating long-acting therapy and monitor HBV DNA in patients who are HBcAb positive and HBsAg negative.

CAB/RPV is a regimen that has proved to be safe and well tolerated, with comparable results in real-world and phase 3 randomized clinical trials [4–7, 12–17]. The SOLAR study evaluated weight, BMI, and body composition measurements in 454 participants switching to CAB/RPV and 227 continuing on BIC/F/TAF in the first year of follow-up; changes in metabolic parameters were minimal and similar between the 2 groups, and there were no differences in the proportions of PWH with metabolic syndrome or insulin resistance [28].

In people switching to long-acting treatment enrolled in the SCohoLART study, HDL levels slightly increased; data on lipid profile in 2-drug regimens are often conflicting, showing either a decrease or an increase in HDL-C with treatment switching, depending on the study [29–32]. The simplification from 3 to 2 drugs in >60% of participants and the absence of drugs known to be associated with the development of dyslipidemia in long-acting therapy may have led to the improvement in lipid profile; however, longer follow-up is needed to confirm these changes over time and assess potential cardiovascular risk modification.

We also observed a decrease in serum creatinine and a consequent increase in eGFR after the switch to long-acting therapy. CAB does not appear to inhibit tubular secretion of creatinine, as it does not lead to inhibition of renal organic cation transporter 2 and multidrug and toxin extrusion transporter 1 [33]. The HPTN083 study, designed to compare the efficacy of CAB with that of tenofovir disoproxil fumarate/F for HIV preexposure prophylaxis, showed that CAB alone could reduce serum creatinine [34], and our study confirmed that combining CAB with RPV also results in this change.

One study limitation is the lack of assessment of CAB concentrations (data on RPV concentrations not shown), which would also allow us to better investigate the underlying causes of VFs. In addition, our cohort mainly included men with a good immunological profile. These results may not be generalizable to other populations, such as women or PWH with more advanced immunosuppression.

In conclusion, the 1-year cumulative probability of TD with CAB/RPV was quite low, and TDs were mostly due to ISRs. During the study follow-up, VFs were rare and must be managed with great care on a case-by-case basis. Based on our findings, switching to long-acting therapy is also a valid option in people with a long history of HIV infection and prolonged exposure to ART.

## References

[1] d'Arminio Monforte A, Sabin CA, Phillips A, et al The changing incidence of AIDS events in patients receiving highly active antiretroviral therapy. Arch Intern Med 2005; 165:416–23. 15738371 10.1001/archinte.165.4.416

[2] Smith CJ, Ryom L, Weber R, et al Trends in underlying causes of death in people with HIV from 1999 to 2011 (D:A:D): a multicohort collaboration. Lancet 2014; 384:241–8. 25042234 10.1016/S0140-6736(14)60604-8

[3] Langebeek N, Gisolf EH, Reiss P, et al Predictors and correlates of adherence to combination antiretroviral therapy (ART) for chronic HIV infection: a meta-analysis. BMC Med 2014; 12:142. 25145556 10.1186/s12916-014-0142-1 PMC4148019

[4] Ramgopal MN, Castagna A, Cazanave C, et al Efficacy, safety, and tolerability of switching to long-acting cabotegravir plus rilpivirine versus continuing fixed-dose bictegravir, emtricitabine, and tenofovir alafenamide in virologically suppressed adults with HIV, 12-month results (SOLAR): a randomised, open-label, phase 3b, non-inferiority trial. Lancet HIV 2023; 10:e566–77. 37567205 10.1016/S2352-3018(23)00136-4

[5] Swindells S, Andrade-Villanueva JF, Richmond GJ, et al Long-acting cabotegravir and rilpivirine for maintenance of HIV-1 suppression. N Engl J Med 2020; 382:1112–23. 32130809 10.1056/NEJMoa1904398

[6] Overton ET, Richmond G, Rizzardini G, et al Long-acting cabotegravir and rilpivirine dosed every 2 months in adults with HIV-1 infection (ATLAS-2 M), 48-week results: a randomised, multicentre, open-label, phase 3b, non-inferiority study. Lancet 2021; 396:1994–2005. 33308425 10.1016/S0140-6736(20)32666-0

[7] Orkin C, Arasteh K, Górgolas Hernández-Mora M, et al Long-acting cabotegravir and rilpivirine after oral induction for HIV-1 infection. N Engl J Med 2020; 382:1124–35. 32130806 10.1056/NEJMoa1909512

[8] European Medicines Agency. Vocabria (cabotegravir). An overview of Vocabria and why it is authorised in the EU. Available at: https://www.ema.europa.eu/en/documents/overview/vocabria-epar-medicine-overview_en.pdf. Accessed 14 February 2024.

[9] Cabenuva—US prescribing information. Available at: https://www.accessdata.fda.gov/drugsatfda_docs/label/2021/212888s000lbl.pdf. Accessed 14 February 2024.

[10] Vocabria EU summary of product characteristics. Available at: https://www.ema.europa.eu/en/documents/product-information/vocabria-epar-product-information_en.pdf. Accessed 14 February 2024.

[11] Rekambys EU summary of product characteristics. Available at: https://www.ema.europa.eu/en/documents/product-information/rekambys-epar-product-information_en.pdf. Accessed 14 February 2024.

[12] Sension MG, Brunet L, Hsu RK, et al Cabotegravir + rilpivirine long-acting injections for HIV treatment in the US: real world data from the OPERA cohort. Infect Dis Ther 2023; 12:2807–17. 37966701 10.1007/s40121-023-00890-2 PMC10746614

[13] Eron JJ, Sarkar S, Frick AJ, et al Real-world utilization of cabotegravir + rilpivirine in the US: data from Trio Health Cohort [abstract 625]. Presented at: 31st Conference on Retroviruses and Opportunistic Infections (CROI); 3–6 March 2024; Denver, CO.

[14] Swindells S, Lutz T, Van Zyl L, et al Week 96 extension results of a phase 3 study evaluating long-acting cabotegravir with rilpivirine for HIV-1 treatment. AIDS 2022; 36:185–94. 34261093 10.1097/QAD.0000000000003025 PMC8711605

[15] Jaeger H, Overton ET, Richmond G, et al Long-acting cabotegravir and rilpivirine dosed every 2 months in adults with HIV-1 infection (ATLAS-2 M), 96-week results: a randomised, multicentre, open-label, phase 3b, non-inferiority study. Lancet HIV 2021; 8:e679–89. 34648734 10.1016/S2352-3018(21)00185-5

[16] Orkin C, Oka S, Philibert P, et al Long-acting cabotegravir plus rilpivirine for treatment in adults with HIV-1 infection: 96-week results of the randomised, open-label, phase 3 FLAIR study. Lancet HIV 2021; 8:e185–96. 33794181 10.1016/S2352-3018(20)30340-4

[17] Orkin C, Bernal Morell E, Tan DHS, et al Initiation of long-acting cabotegravir plus rilpivirine as direct-to-injection or with an oral lead-in in adults with HIV-1 infection: week 124 results of the open-label phase 3 FLAIR study. Lancet HIV 2021; 8:e668–78. 34656207 10.1016/S2352-3018(21)00184-3

[18] Thoueille P, Saldanha SA, Schaller F, et al Real-world trough concentrations and effectiveness of long-acting cabotegravir and rilpivirine: a multicenter prospective observational study in Switzerland. Lancet Reg Health Eur 2023; 36:100793. 38162253 10.1016/j.lanepe.2023.100793 PMC10757247

[19] Latham C, Urbaityte R, Sutton K, et al HIV-1 RNA blips and low-level viral replication: SOLAR (CAB + RPV LA vs BIC/FTC/TAF) [abstract 627]. Presented at: 31st Conference on Retroviruses and Opportunistic Infections (CROI); 3–6 March 2024; Denver, CO.

[20] Orkin C, Schapiro JM, Perno CF, et al Expanded multivariable models to assist patient selection for long-acting cabotegravir + rilpivirine treatment: clinical utility of a combination of patient, drug concentration, and viral factors associated with virologic failure. Clin Infect Dis 2023; 77:1423–31. 37340869 10.1093/cid/ciad370 PMC10654860

[21] Sension M, Hsu RK, Fusco JS, et al Real-world effectiveness of long-acting cabotegravir + rilpivirine in virologically suppressed treatment-experienced individuals: two years of data from the OPERA® cohort [abstract 1026]. Presented at: IDWeek 2023; 11–14 October 2023; Boston, MA.

[22] Champredon D, Bellan SE, Delva W, et al The effect of sexually transmitted co-infections on HIV viral load amongst individuals on antiretroviral therapy: a systematic review and meta-analysis. BMC Infect Dis 2015; 15:249. 26123030 10.1186/s12879-015-0961-5 PMC4486691

[23] Muccini C, Crowell TA, Pinyakorn S, et al Brief report: syphilis incidence and effect on viral load, CD4, and CD4/CD8 ratio in a Thai cohort of predominantly men who have sex with men living with HIV. J Acquir Immune Defic Syndr 2021; 86:219–23. 33433124 10.1097/QAI.0000000000002542

[24] van Welzen BJ, Van Lelyveld SFL, Ter Beest G, et al Virological failure after switch to long-acting cabotegravir and rilpivirine injectable therapy: an in-depth analysis. Clin Infect Dis 2024; 11:ciae016. 10.1093/cid/ciae016 PMC11259215 38207125

[25] Wensing AM, Calvez V, Ceccherini-Silberstein F, et al 2019 update of the drug resistance mutations in HIV-1. Top Antivir Med 2019; 27:111–21. 31634862 PMC6892618

[26] Xu HT, Colby-Germinario SP, Asahchop EL, et al Effect of mutations at position E138 in HIV-1 reverse transcriptase and their interactions with the M184I mutation on defining patterns of resistance to nonnucleoside reverse transcriptase inhibitors rilpivirine and etravirine. Antimicrob Agents Chemother 2013; 57:3100–9. 23612196 10.1128/AAC.00348-13 PMC3697388

[27] Denyer RV, Tate JP, Benator DA, et al Hepatitis B reactivation in persons with HIV with positive hepatitis B core antibody after switching to antiretroviral therapy without hepatitis B activity [abstract 1026]. Presented at: IDWeek 2023; 11–14 October 2023; Boston, MA.

[28] Stan DH, Antinori A, Eu B, et al Weight and metabolic changes with cabotegravir + rilpivirine long-acting or bictegravir/emtricitabine/tenofovir alafenamide [abstract 146]. Presented at: Conference on Retroviruses and Opportunistic Infections (CROI); 19–23 February 2023; Seattle, WA.

[29] Maggiolo F, Gulminetti R, Pagnucco L, et al Lamivudine/dolutegravir dual therapy in HIV-infected, virologically suppressed patients. BMC Infect Dis 2017; 17:215. 28302065 10.1186/s12879-017-2311-2 PMC5356275

[30] Hidalgo-Tenorio C, López Cortés L, Gutiérrez A, et al DOLAMA study: effectiveness, safety and pharmacoeconomic analysis of dual therapy with dolutegravir and lamivudine in virologically suppressed HIV-1 patients. Medicine (Baltimore) 2019; 98:e16813. 31393412 10.1097/MD.0000000000016813 PMC6708975

[31] Baldin G, Ciccullo A, Rusconi S, et al Long-term data on the efficacy and tolerability of lamivudine plus dolutegravir as a switch strategy in a multi-centre cohort of HIV-1-infected, virologically suppressed patients. Int J Antimicrob Agents 2019; 54:728–34. 31521809 10.1016/j.ijantimicag.2019.09.002

[32] Tan M, Johnston S, Nicholls J, Gompels M. Dual therapy with renally adjusted lamivudine and dolutegravir: a switch strategy to manage comorbidity and toxicity in older, suppressed patients? HIV Med 2019; 20:634–7. 31338933 10.1111/hiv.12781 PMC6790693

[33] ViiV Healthcare . Global data sheet for cabotegravir (PrEP). Version 01. 1 July 2021.

[34] Landovitz RJ, Donnell D, Clement ME, et al Cabotegravir for HIV prevention in cisgender men and transgender women. N Engl J Med 2021; 385:595–608. 34379922 10.1056/NEJMoa2101016 PMC8448593

